# Regional brain functions in the resting state indicative of potential differences between depression and chronic pain

**DOI:** 10.1038/s41598-017-03522-1

**Published:** 2017-06-07

**Authors:** Atsuo Yoshino, Yasumasa Okamoto, Mitsuru Doi, Naofumi Otsuru, Go Okada, Masahiro Takamura, Naho Ichikawa, Satoshi Yokoyama, Hidehisa Yamashita, Shigeto Yamawaki

**Affiliations:** 1 Department of Psychiatry and Neurosciences, Division of Frontier Graduate School of Biomedical Sciences, Hiroshima University, 1-2-3 Kasumi, Minami-ku, Hiroshima 734-8551 Japan; 2 Department of Dental Anesthesiology, Hiroshima University, 1-2-3 Kasumi, Minami-ku, Hiroshima 734-8551 Japan; 3 Department of Physical Therapy, Niigata University of Health and Welfare, 1398 Shimamichou, Kita-ku, Niigata 950-3198 Japan

## Abstract

Complex relationships between depression and chronic pain have been reported in previous studies. However, only a few neuroimaging studies have investigated similarities and differences in neural systems underlying them. We examined the brain functions in the resting state of 43 patients with depression, 41 patients with chronic pain (somatoform pain disorder) and 41 healthy controls, by using regional homogeneity (ReHo) and functional connectivity analysis. Depressive symptoms were assessed by using the Beck Depression Inventory-Second Edition (BDI-II). ReHo values for the dorsolateral prefrontal cortex (DLPFC) significantly decreased for chronic pain patients, and functional connectivity between the DLPFC and thalamus decreased only for these patients. These findings are indicative of distinct brain functions related to depression and chronic pain. Understanding these differences would further elucidate the pathophysiology of these conditions.

## Introduction

A number of epidemiological studies have shown that both depression and chronic pain lead to decreased productivity, social disability, increased suicide rates and higher health care cost^1–4^. The association between depression and chronic pain has been supported by previous studies, including biological studies on neuroplastic, neurochemical, electrophysiological and hormonal variables, and psychological studies on pessimism and low self-esteem^5, 6^. Furthermore, different randomized controlled trials have reported that antidepressants have beneficial effects on both depressive symptoms and pain perception^7, 8^. Thus, it appears that depression and chronic pain might have certain commonalities.

Subtle differences between chronic pain and depressive patients have been reported. For instance, certain experimental studies on pain perception using thermal, or electrical stimuli have shown that chronic pain patients exhibit higher pain sensitivity than healthy controls^9–11^. However, other studies of depressive patients have indicated that they were less likely to perceive pain stimuli compared to controls^12, 13^. To our knowledge, there is only one study that has directly compared pain perception between depressive and chronic pain patients. Normand *et al*. examined potential differences in experimentally induced pain perception and diffuse noxious inhibitory control efficacy (e.g. “pain inhibits pain” phenomenon) between depressive and chronic pain patients by using a tonic thermal test and a cold pressor test^14^. They reported that chronic pain could be distinguished from depression by pain ratings during the cold pressor test, which might be related to the pain inhibition system. Another study has suggested that efficacy of antidepressants against depressive symptoms and pain, including the dose and onset of efficacy, might be based on independent mechanisms^15^. Moreover, path analysis has suggested that analgesic effects of antidepressants in chronic pain patients might be separately caused by a direct analgesic effect and an antidepressant effect^16^. They have indicated that 80% and over of the change in pain intensity in chronic pain patients could be ascribed to a direct analgesic effect of antidepressants, with the remaining effect affected by an antidepressant effect.

Various brain regions involved in the processing of sensation, emotion, cognition, and nociception are associated with the neuropathology of both depression and chronic pain^17–19^. The amygdala, hippocampus, insula, anterior cingulate cortex (ACC) and the prefrontal cortex (PFC) show alterations in depression^19^. On the other hand, the somatosensory cortex, thalamus, amygdala, hippocampus, insula, ACC and the PFC are involved in chronic pain^17^. Various studies of the linkage between depression and chronic pain have reported the ACC to be a critical region^20, 21^, and many neuroimaging studies have suggested that the ACC is significantly involved in depression or chronic pain^17–19, 22, 23^. On the other hand, based on the above-mentioned studies, distinctive brain regions involved in chronic pain would appear to be the somatosensory cortex and thalamus. Moreover, pain-related catastrophizing, that is mainly associated with etiology of chronic pain, and depression differed in the ways in which they impacted on pain experiences such as pain prediction^24^. Pain-related catastrophizing is closely related to activity in the dorsolateral prefrontal cortex (DLPFC)^25^, and of the PFC structures, the DLPFC seems to show the most differentiation between depression and chronic pain, playing different roles in each of these phenomena. Based on these points, we hypothesized that the activities of the DLPFC, thalamus and somatosensory cortex would be different between depression and chronic pain, and that the ACC would be a common, activated region in depressive and chronic pain patients, but not in healthy participants.

Recent evidence indicates that resting-state functional magnetic resonance imaging (R-fMRI) might be useful for investigating human cognitions, behaviors, emotions and somatic sensations^26, 27^. It is known that regional homogeneity (ReHo) has been shown to be sufficient to measure the local temporal synchronization of the time series of nearest neighbors during the resting state^28, 29^, and the functional connectivity method can manifest longer inter-regional changes^19, 28^. Previous ReHo or functional connectivity studies have suggested that these techniques were useful for increasing our understanding of neuropathology related to mental disorders or chronic pain^27, 30–32^. Furthermore, coordinating the ReHo method in the seed-based functional connectivity analysis may facilitate the sensitivity of each analysis and reduce the uncertainty of seed extraction^33^, and more sensitive analyses could contribute to further elucidation of the complex neurocircuitry underlying depression and chronic pain.

In order to test the present hypotheses, we examined underlying resting state neural abnormalities of 43 depressive patients, 41 chronic pain patients, and 41 healthy controls using ReHo (local activity) and functional connectivity methods (long-distance connectivity). We conducted a one-way analysis of covariance (ANCOVA) with Beck Depression Inventory-Second Edition (BDI-II) scores as a continuous factor, BDI-II scores * group as the interaction and age and gender as covariates of no interest.

## Results

### Characteristics of participants

Detailed demographic and clinical characteristics of participants are presented in Table 1. There were no significant differences in age, or gender among the groups (*p* = 0.46 for age and *p* = 0.38 for gender). A one-way ANOVA revealed a significant main effect of BDI-II scores, *F* (2, 122) = 113.8, *p* < 0.001, with BDI-II scores of depressive patients being significantly higher than those of chronic pain patients and the controls (Bonferroni *p* < 0.001). Moreover, BDI-II scores of chronic pain patients were significantly higher than those of the controls (Bonferroni *p* < 0.001).

**Table 1:** Demographic and psychometric variables of patients and controls.

	Depression (n = 43)	Chronic pain (n = 41)	Controls (n = 41)	*F*_score_ or *χ* ^2^_score_
Age	44.4 ± 11.0	47.5 ± 13.4	45.9 ± 9.4	0.7^ns^
Female/Male	23/20	28/13	25/16	1.9^ns^
BDI-II	30.3 ± 8.9	13.2 ± 8.5	5.5 ± 5.3	113.8*
[Psychiatric comorbidity]				
Generalized anxiety disorder	3	2	—	—
Panic disorder	4	0	—	—
Agoraphobia	5	0	—	—
Social anxiety disorder	3	0	—	—
[Medication]				
Antidepressants (n)	38	20	—	—
Anticonvulsants (n)	6	11	—	—
Antipsychotics (n)	9	4	—	—
Minor tranquilizers (n)	21	8	—	—
Analgesics (n)	2	8	—	—

ns = not significant, *p < 0.05 (ANOVA).

BDI-II; Beck Depression Inventory- Second Edition.

### Resting-state regional brain functions in each group

**Figure 1:**
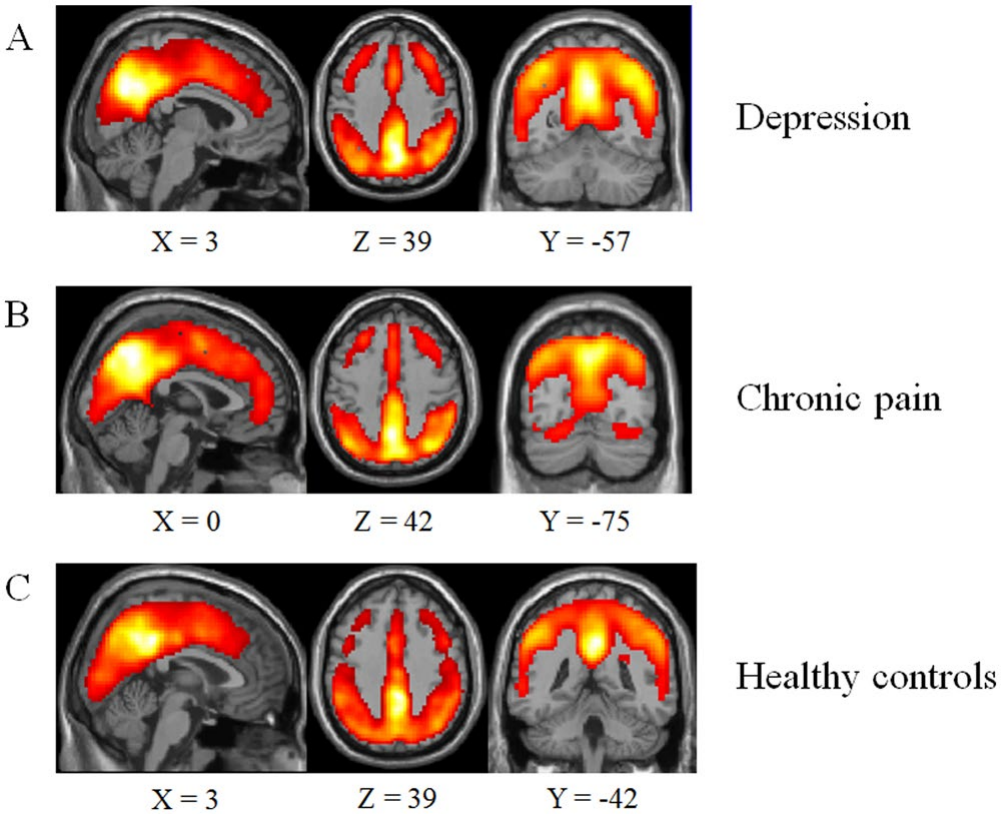
ReHo values for one sample *t*-test (**A**) for patients with depression, (**B**) for patients with chronic pain, and (**C**) for healthy controls.

Regional brain functions of each group are shown in Fig. 1. It can be seen that regions such as the medial temporal lobe, posterior cingulate cortex, postcentral gyrus and precuneus exhibited significantly higher ReHo values during the resting state.

### Between group differences in regional brain functions in the resting state

**Figure 2:**
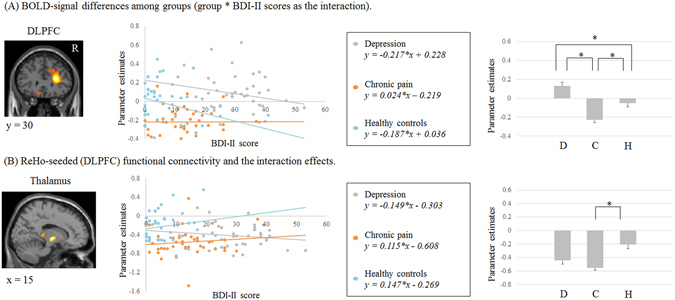
(**A**) Different ReHo values in the DLPFC are shown (*p* (FWE corrected) < 0.05). Scatter-plots illustrate these correlations. *p < 0.01, Bonferroni post hoc test. DLPFC; dorsolateral prefrontal cortex, D; depression, C; chronic pain and H; healthy controls. Each error bar represents one standard error. (**B**) Hypoconnectivity in the DLPFC and thalamus is noted in the chronic pain patients (*p* (FWE corrected) < 0.05). Scatter-plots illustrate these correlations. *p < 0.01, Bonferroni post hoc test. Each error bar represents one standard error.

To examine differences in resting-state regional brain functions among depressive, chronic pain and control groups, we performed an ANCOVA with group as a categorical factor, BDI-II scores as a continuous factor, BDI-II scores * group as the interaction term and age and gender as covariates of no interest, because it is important to assess the implication of depressive state for each group. Significant BDI-II score * group interactions were observed for the DLPFC (Fig. 2A and Table 2: FWE corrected *p* < 0.05).

**Table 2:** BOLD-signal differences among groups (group*BDI-II scores as the interaction).

Brain regions	L/R	x/y/z	z-score	Cluster extent	Bonferroni post hoc
DLPFC	R	24/24/33	4.87	399	Chronic pain < Depression*, Control* Control < Depression*

Voxel level threshold were *p* (uncorrected) < 0.001, and cluster size threshold were *p* (FWE corrected) < 0.05. DLPFC; Dorsolateral Prefrontal Cortex. *p < 0.01.

### DLPFC

After controlling for the effect of BDI-II scores, ReHo values were significantly lower in chronic pain patients than in depressive patients (post hoc test (Bonferroni) *p* < 0.01) and in controls (post hoc test (Bonferroni) *p* < 0.01) (Fig. 2A).

### ReHo-seeded Functional Connectivity

A whole-brain ANCOVA suggested distinctive resting state regional brain functions for the right DLPFC in chronic pain patients, versus depressive patients or controls. We performed ReHo-seeded functional connectivity analysis to assess group differences. We conducted an ANCOVA with group as the categorical factor, BDI-II scores as a continuous factor, BDI-II scores * group as the interaction term, and age and gender as covariates of no interest. For the DLPFC seed, a significant interaction between BDI-II scores and group was observed in the thalamus [x = 15, y = −9, z = −3; *z*-score 4.32, cluster extent 68] (Fig. 2B: FWE corrected *p* < 0.05). After controlled effect of BDI-II scores, functional connectivity values significantly decreased in chronic pain patients than in controls (post hoc test (Bonferroni) *p* < 0.01).

## Discussion

This is the first neuroimaging study conducted with participants in the resting-state to have investigated differences in neural mechanisms between depressive and chronic pain patients. Results indicated that the DLPFC displayed lower ReHo values in chronic pain patients. Additionally, functional connectivity between DLPFC and thalamus was reduced in chronic pain patients.

A significant decrease in ReHo values of the DLPFC, and a reduced functional connectivity between the DLPFC and thalamus was observed in the present study in patients with chronic pain, compared to patients with depression and healthy controls. A decreased functional or structural changes in the DLPFC of patients with chronic pain was also reported in previous studies^30, 34–36^. Our results corroborated these previous studies. It has been demonstrated that the DLPFC has greater control over pain perception, including its sensory and affective dimensions^37^. The present study showed a significant change in the DLPFC (BA46) of patients with depression. A number of previous studies conducted in the resting state showing abnormal DLPFC activity in depressive patients have indicated the involvement of the left DLPFC and the Brodmann area (BA 9)^38^. Abnormal DLPFC activation in this study was mainly indicated on the right side and BA 46, which might be suggestive of specific changes in patients with chronic pain.

Lorenz *et al*. have proposed the important role played by the DLPFC (BA 9 and 46) in active manipulation of pain perceptions through the modulation of cortico-subcortical pathways^37^. The current study also demonstrated that the DLPFC was differently connected with the thalamus in the groups that were investigated. Many studies have demonstrated effective interactions between the DLPFC and midbrain, thalamic, striatal and cingulate structures of the limbic system^26, 39, 40^. Tran *et al*. have reported that thalamocortical mechanisms including the functional connectivity between the DLPFC and thalamus contributed to pain modulatory effects^40^. Previous studies on chronic pain have also suggested an abnormal connectivity between the PFC and thalamus^41–43^. The thalamus is also considered to play an important role in pain processing^18^. The present study speculates that poor functional connectivity between the thalamus and DLPFC, specific only to chronic pain patients, would be based on an abnormality of a top-down mode through the descending pathway from the PFC of inhibiting neuronal activities along the ascending pain pathway including the thalamus.

We have not found similarities in the ACC in depressive and chronic pain patients. Neuroimaging studies have shown that depression was mainly related to abnormal activations in Brodmann’s area 25 and that chronic pain was strongly linked to the dorsal ACC, such as the Brodmann’s area 24^44, 45^. It is possible that certain other localizations between depression and chronic pain influenced the present results.

The present results were also dissimilar to our hypothesis that the somatosensory cortex would show more abnormal brain functions in patients with chronic pain. Many previous studies implicate the key brain limbic and cortical structures engaged in the pain-related cognitive, emotional and behavioral controls as the cause of chronic pain, which might have been associated with the result.

The results of the present study are constrained by several limitations. Our exclusion criteria for participants did not include all possible treatment effects that might influence depressive states, and pain perceptions of patients, such as the use of antidepressants. We also could not rule out all treatment effects on brain functions that were observed in this study. Furthermore, many clinical characteristics such as pain perception and anxiety were not assessed. Finally, we excluded patients that have both depression and chronic pain. We believe that the strategy is useful to identify factors that are specific to chronic pain vs. depression, but it has also a limitation related to generalizability and clinical relevance.

In conclusion, we investigated commonalities and differences in brain regions of depressive and chronic pain patients. The results suggest that the DLPFC and thalamus are associated with the dysfunction of pain-controlling networks specific to chronic pain. It is suggested that further comparisons be conducted to determine the similarities and differences in pathological conditions of depressive and chronic pain patients, which might contribute to the development of clinical treatment, among others.

## Materials and Methods

### Participants

Participants were 43 patients with major depressive disorder (MDD, 23 women, mean age = 44.4 ± 11.0 years), diagnosed according to DSM-IV-TR criteria, 41 patients with chronic pain (28 women, mean age = 47.5 ± 13.4 years), diagnosed according to DSM-IV-TR criteria as having SPD, and 41 control participants (25 women, mean age = 45.9 ± 9.4 years). All participants were Japanese people that provided written informed consent for participating in the present study. The study was designed and conducted according to a protocol approved by the ethics committee of Hiroshima University. The criterion for inclusion in the study was a diagnosis of MDD and SPD, as established by a psychiatrist with more than 10-years of experience in using the Structured Clinical Interview for DSM-IV (SCID) for patients with SPD^46^, or in using the Mini-international neuropsychiatric interview for patients with MDD^47^. SPD is defined as the occurrence of one or more physical complaints for which appropriate medical evaluation reveals no explanatory physical pathology or pathophysiologic mechanism, or when such a pathology is present, the physical complaints or resulting impairment are grossly in excess of what would be expected from the physical findings^46^, and SPD is known to often occur in patients with chronic pain^48^. Details of psychiatric comorbidities and medication are shown in Table 1. Exclusion criteria for depression and chronic pain included the following: (1) HIV-related pain and cancer pain, because these are associated with malignant diseases which entail a different symptom trajectory than in chronic pain, (2) difficulty in understanding the purpose of the study (e.g. presence of dementia, delirium, or psychosis), (3) organic brain disorders (e.g. cerebral hemorrhage, infarction), (4) schizophrenia, bipolar affective disorders, or seizure disorders that are inadequately controlled by medication, (5) current substance abuse and (6) use of opioid medication. Furthermore, exclusion criteria for chronic pain included current MDD. Also, exclusion criteria for depression included a BDI-II score under 10, and current treatment for chronic pain. Normal control participants were recruited from a non-clinical population. Control participants neither complained of problems related to chronic pain, nor had a history of psychiatric disorders.

### Clinical assessments

BDI-II was used to assess depressive symptoms^49^.

### fMRI acquisition

The fMRI procedure was performed using a Magnex Eclipse 3 T Power Drive 250 (Siemens, Munich, Germany). A time course series of 120 scans was acquired using T2*-weighted, gradient echo, echo planar imaging (EPI) sequences. Each volume consisted of 28 slices, with a slice thickness of 4 mm with no gaps, which covered the entire cerebral and cerebellar cortices. The time interval between two successive acquisitions of the same image (TR) was 3000 ms, the echo time (TE) was 46 ms, and the flip angle was 90°. The field of view (FOV) was 256 mm, and the matrix size was 64 × 64, giving voxel dimensions of 4 mm × 4 mm × 4 mm. Scan acquisition was synchronized to the onset of each trial. After functional scanning, structural scans were acquired using a T1-weighted gradient echo pulse sequence (TR = 2160 ms; TE = 3.93 ms; flip angle = 15°; FOV = 256 mm; voxel dimensions of 1 mm × 1 mm × 1 mm) to facilitate localization. After scanning, participants were asked whether they had kept their eye closed and whether they had remained awake during the scan. All participants confirmed that they had kept their eyes closed and remained awake.

### Analysis of functional imaging data

The first 10 images were discarded to ensure steady-state MRI signals during acclimation of participants and images collected after acclimation were further analyzed. Image preprocessing was conducted using Statistical Parametric Mapping (SPM8) software (Wellcome Department of Cognitive Neurology, London, UK). First, slice timing and head movement corrections were conducted. Then, translation (mm) and rotation (degrees) values were obtained at each time point. All participants had less than 1.5 mm maximum displacement in the x, y, and z axes, and less than 1.5° angular motion during the scan. The images were then spatially normalized to the Montreal Neurological Institute (MNI) functional template (resampling voxel size = 3 mm × 3 mm × 3 mm).

After preprocessing in SPM8 and a motion scrubbing procedure^50^, linear trends were removed and ReHo analysis was conducted using the R-fMRI data analysis toolkit (REST, http://restfmri.net/forum/), version 1.6. The ReHo analysis procedure was conducted according to the method described by Zang *et al*.^29^. ReHo was defined as the Kendall’s coefficient of concordance (KCC)^51^ of the time series for a given voxel with those of its nearest neighbors. Cubic clusters of 27 voxels were used for ReHo value of every cubic cluster to the central voxel. The square root was calculated at each frequency of the power spectrum. The sum of amplitudes across 0.01–0.08 Hz was divided by the amplitude across the entire frequency range. All ReHo maps were smoothed with a Gaussian filter of 9 mm full-width half-maximum kernel to manage the anatomical variability that was not compensated for by spatial normalization, and to improve the signal-to-noise ratio. Group-level analyses of the ReHo maps were conducted using SPM8. To separately detect regional brain functions in each group (depression, chronic pain and healthy controls), a one-way analysis of covariance (ANCOVA) with group as the categorical factor, BDI-II scores as a continuous factor, BDI-II scores * group as the interaction and age and gender as covariates of no interest was conducted. In the whole brain analysis, cluster size thresholds were *p* (FWE corrected) < 0.05. Voxel parameter estimates of main effects were examined using post-hoc Bonferroni multiple comparisons performed by using SPSS version 16.0. The spatial coordinates provided by SPM8, which are in MNI brain space were converted to spatial coordinates of the Anatomical Automatic Labeling (AAL) atlas using the MarsBar SPM Toolbox (http://www.sourceforge.net/projects/marsbar).

Furthermore, functional connectivity analysis of ReHo-based seeds was conducted using the R-fMRI data analysis toolkit (REST, http://restfmri.net/forum/) version 1.6, to examine interactions between brain regions related to the experimental paradigm. To perform functional connectivity analysis, the first eigenvariate time series of brain regions identified as being activated by the previous analyses was extracted as a ROI. For each participant, the mean ROI time series were computed for reference time course. A whole brain analysis for the ROI was then conducted. Finally, Fisher’s *z*-transformation was applied to improve the normality of the correlation coefficients^28^. To detect between group differences, a one-way analysis of covariance (ANCOVA) with group as the categorical factor, BDI-II scores as a continuous factor, BDI-II scores * group as the interaction and age and gender as a covariate of no interest was conducted. Cluster size thresholds were *p* (FWE corrected) < 0.05.
